# Nuts and Human Health Outcomes: A Systematic Review

**DOI:** 10.3390/nu9121311

**Published:** 2017-12-02

**Authors:** Rávila Graziany Machado de Souza, Raquel Machado Schincaglia, Gustavo Duarte Pimentel, João Felipe Mota

**Affiliations:** Clinical and Sports Nutrition Research Laboratory (LABINCE), Faculty of Nutrition, Goiás Federal University, Goiânia 74605-080, Brazil; ravilagraziany@gmail.com (R.G.M.d.S.); raquelms@outlook.com (R.M.S.); gupimentel@yahoo.com.br (G.D.P.)

**Keywords:** tree nuts, health, obesity, body weight, cancer

## Abstract

There has been increasing interest in nuts and their outcome regarding human health. The consumption of nuts is frequently associated with reduction in risk factors for chronic diseases. Although nuts are high calorie foods, several studies have reported beneficial effects after nut consumption, due to fatty acid profiles, vegetable proteins, fibers, vitamins, minerals, carotenoids, and phytosterols with potential antioxidant action. However, the current findings about the benefits of nut consumption on human health have not yet been clearly discussed. This review highlights the effects of nut consumption on the context of human health.

## 1. Introduction

There is increasing interest in nut consumption and human health outcomes [1]. Nuts are commonly consumed in the Mediterranean diet, and their consumption has been recommended to populations all over the world [2]. Tree nuts, such as almonds, hazelnuts, cashew nuts, Brazil nuts, macadamias, walnuts, and pistachios, as well as legume seeds, such peanuts, are nutrient-dense foods each with a unique composition. In general, these foods contain healthy monounsaturated (MUFA) and polyunsaturated (PUFA) fatty acid profiles; protein; soluble and insoluble fibers; vitamins E and K; folate; thiamine; minerals such as magnesium, copper, potassium, and selenium; and substances such as zanthophyll carotenoids, antioxidants, and phytosterols compounds, with recognized benefits to human health [3,4,5].

Prior reviews and epidemiological and/or clinical trials have suggested that regular nut consumption has beneficial impact on health outcomes, such as obesity [6], hypertension [7], diabetes mellitus [8], and cardiovascular diseases [3], with reduction in mediators of chronic diseases such as oxidative stress, inflammation, visceral adiposity, hyperglycemia, insulin resistance, endothelial dysfunction, and metabolic syndrome [9]. Furthermore, several prospective studies and clinical trials have reported beneficial effects after nut consumption. However, the current findings about the main benefits of the consumption of each nut type on human health have not yet been discussed. In addition, main nutritional components and practical implications regarding nut consumption in humans need to be better clarified. Therefore, in this systematic review, we highlight the effects of nut consumption on human health.

## 2. Method

**Figure 1:**
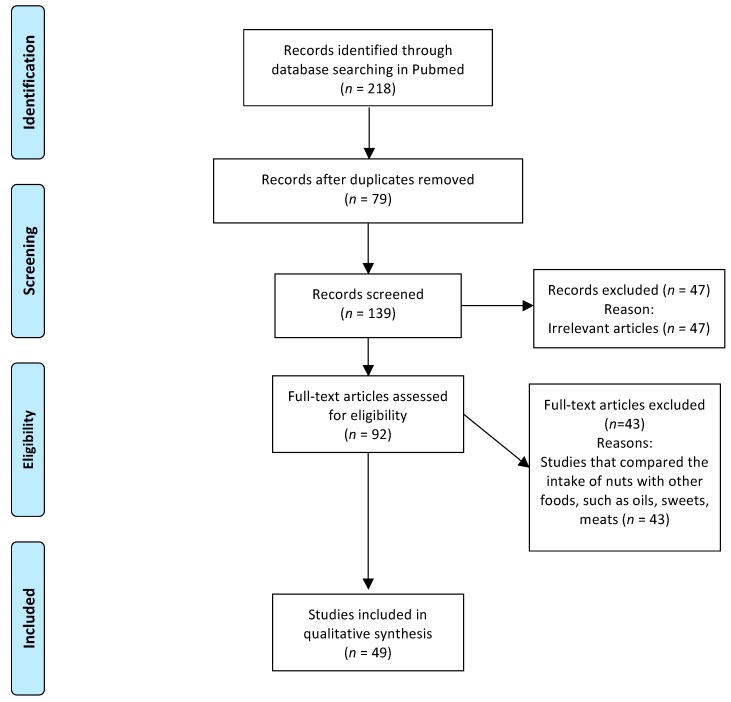
Flow diagram of the structured literature review.

We used the PRISMA (Preferred Reporting Items for Systematic Reviews and Meta-Analyses) method to systematically review the articles that assessed the effects of nut consumption on human health. This systematic review was registered in International prospective register of systematic reviews (PROSPERO) with the number CRD42017077466. We conducted the review using the electronic database PubMed^®^/MEDLINE^®^ (https://www.ncbi.nlm.nih.gov/pubmed/) and by searching clinical trials published from 2007 to July 2017 that were published in journals with an impact factor (according to the Journal of Citation Reports) of ≥1.0. The criteria to define a “clinical trial” was based on studies with humans that were prospectively assigned to one or more interventions (which may include placebo or other control groups), and with the aim to evaluate the effects of interventions with nuts on human health. Since the Medical Subject Headings (MeSH) is the National Library of Medicine (NLM) controlled vocabulary thesaurus used for indexing articles for PubMed^®^/MEDLINE^®^ we used it to select the index terms. The search yielded the following keywords: almonds, Brazil nut, cashew nut, hazelnut, macadamia, peanuts, pistachio, walnut, tree nuts, ground nut, diabetes, obesity, dyslipidemia, heart disease, cancer, body weight, digestion, food intake, human health, inflammation, and oxidative stress. The Boolean operators “and”, “or”, and “and not” were used to combine the terms used in the literature review. The initial search consisted of screening titles and abstracts, whereas the second step consisted of reviewing full-text articles to confirm the study selection. The information extracted from each individual study was as follows: year of publication, objective, nut type, portion size, and relationship to human health. The search strategy and the total of evaluated and selected studies are shown in Figure 1. As a result of the search, 49 studies were selected. In addition, this review included the citation of nine articles that were used to define the scientific terms and discuss the results.

## 3. Results

### 3.1. Almonds

Almonds can improve human health when consumed in small (10 g/day) [10] or large portions (100 g/day) [11]. Results of clinical trials suggest potential effect on glycemic control and lipid metabolism, reducing postprandial blood glucose [12], serum insulin, insulin resistance [13], and lipid peroxidation [14], and improving high-density lipoprotein (HDL-c) concentrations [15]. Almonds could even be a coadjuvant food in the treatment of individuals who do not wish to take or cannot use large doses of statins [11]. In addition, almond consumption in isocaloric diets may help control satiety [12,15] and improve cognitive function in individuals with a low-calorie diet prescription [16]. In spite of the high caloric density of almonds, studies do not show weight gain in individuals eating diets with lower or higher amounts of almonds [15,17].

The consumption of almonds at snack time reduces postprandial blood glucose and increases satiety in individuals with type 2 diabetes risk. This conclusion was obtained by evaluating the consumption of 43 g/day of almonds over a four-week period at different times of the day, following meals such as breakfast and lunch or alone as a morning or afternoon snack. The groups that consumed almonds at snack time had a greater reduction in the sensation of hunger and serum glucose concentrations. Despite an intake of 250 kcal of almonds per day, there was no weight gain among the participants evaluated [12]. According to the authors, the satiety sensation may compensate for the energy surplus, and it is known that the energy absorption from almonds is not totally efficient due to the nutritional composition of this food, such as the high amount of fiber [12]. According to Gebauer et al. (2016), the amount of calories absorbed from almonds is dependent of the form in which they are consumed [18]. The metabolizable energy of whole natural almonds, whole roasted almonds, and chopped almonds was significantly lower than predicted with Atwater factors. In addition, the metabolizable energy of whole natural almonds was lower than whole roasted almonds. The authors suggested that this may occur due to lower hardness of whole roasted when compared to whole natural almonds, and to whole natural almonds fracturing into fewer, larger particles, thus inhibiting the release of lipids [18].

Daily intake of an even greater amount of almonds (1440 kJ, equivalent to 344 kcal or 60 g) for 10 weeks did not promote weight gain in overweight and obese women, and this was attributed to a spontaneous reduction of caloric intake from other dietary sources [17]. On the other hand, a randomized trial that evaluated the effects of an almond-enriched low-calorie diet (28 g/day of almonds) observed a slightly lower weight loss but a greater improvement in the lipid profile when compared to a control low-calorie diet group after six months of intervention. However, no differences were observed between the groups after 18 months of intervention [19].

Similarly, the addition of 100 g of almonds daily for four weeks did not increase body weight, despite higher daily energy intake, but it reduced non–HDL-c in patients with statin therapy and elicited beneficial trends in other lipoprotein parameters, reducing cardiovascular risk. Although almonds are energy-dense, some nutrients such as protein, unsaturated fat, and fiber cause satiation, limiting the consumption of additional calories. Thus, almonds may be especially appealing to patients unwilling or unable to take high statin doses [11].

In another study with diabetic individuals, 60 g/day of almonds (20% of the total energy intake) for four weeks reduced serum insulin concentrations by 4.1% and the insulin resistance index (homeostasis model assessment, HOMA-IR) by 9.2% compared to the control group. In addition, the study observed enhancement in the plasma α-tocopherol level and reduction of total cholesterol (TC), low-density lipoprotein (LDL-c), and the ratio of LDL-c to HDL-c by 6.0%, 11.6%, and 9.7%, respectively. Apolipoprotein (Apo) B levels, the Apo B/Apo A-1 ratio, and non-esterified fatty acids (NEFA) also decreased by 15.6%, 17.4%, and 5.5%, respectively [13]. It was believed that changes in lipid profile were caused by almonds’ fatty acid composition.

Furthermore, substituting a high-carbohydrate snack (i.e., muffin) with one serving of almonds (~42.5 g) for six weeks may be a simple dietary strategy to prevent the onset of cardio-metabolic diseases. Substitution with almonds reduced abdominal and leg adiposity deposits without differences in body weight [15]. Even in small quantities (10 g/day), almonds increased HDL-c and decreased TC, triglycerides, LDL-c, and very low-density lipoprotein (VLDL-c) in coronary artery disease patients [10]. With energy restriction, almond consumption is observed to improve body composition and fat loss in the truncal area in compliant overweight or obese adults [16].

Other health benefits include reduction in biomarkers of lipid peroxidation, such as malondialdehyde (MDA) and urinary isoprostanes in older hyperlipidemic subjects [14]. An almond-enriched diet can also promote changes in post-lunch dip and long-term cognitive function in energy-restricted overweight and obese adults [20]. Another benefit found was the improvement in diet quality and gut microbiota, especially in children; however, without alterations in immune status or in phylum and family level, genus-level changes occurred with nut intake, especially in children [21]. Therefore, several studies have demonstrated that almond intake (10–100 g/day) is associated with improvement in glucose homeostasis and lipid metabolism. Moreover, the high fiber consumption may be the main factor related to the increase in satiety and weight control. The benefits of almond intake to human health include reduction of cardiovascular risk [10,11], with evident benefits for diabetic [12,13], hyperlipidemic [14] and obese individuals [16,17,20,21]. Studies on almonds and health outcomes are summarized in Table 1.

### 3.2. Walnuts

Similar to almond consumption, the beneficial effects on human health of walnut intake may be seen with acute ingestion [22], as well as with chronic consumption [12] (Table 2). Studies with walnut consumption between 21 and 75 g/day [23,24,25,26] have shown a reduction in cardiovascular risk [27] with an improvement in the lipid profile [22] and endothelial function [28]. Walnut intake seems to be a strategy to improve diet quality, adjust dietary nutrients, and replace less nutritious foods in the diet. For example, the inclusion of 75 g/day of walnuts in the diet resulted in a reduction of energy intake and provided beneficial nutrients, such as PUFA, omega-3 fatty acids, and dietary fiber [26].

The main effects of consumption of 30 g of walnuts in a low-fat diet occur by increased dietary PUFA in the first three to six months, with greater reductions in fasting insulin levels (*p* = 0.046) and body weight (*p* = 0.028) [29]. Walnuts, as part of a mixed diet in healthy adults, provide less available energy (21%) than predicted by the Atwater factors, which may help explain why consumers of nuts do not gain excessive weight [30]. Another potential mechanism that could explain these findings is that nuts alter satiation. A clinical trial showed an increase in satiation and a sense of fullness within three to four days after beginning an isocaloric diet containing 48 g/day of walnuts; however, no effects were observed on resting metabolic expenditure, insulin resistance, or hormones known to mediate satiety in subjects with metabolic syndrome [31].

In type 2 diabetic individuals, the consumption of an ad libitum diet enriched with 56 g/day of walnuts for eight weeks improved endothelial function compared to an ad libitum diet without walnuts [24]. The effects of walnuts on markers of atherosclerosis and coronary heart disease risk were also studied by Canales et al. (2011) [32]. The authors offered walnut paste-enriched meat products to subjects at increased cardiovascular risk and found higher activity of paraoxonase, a protein that exerts protective cardiovascular effects and lower levels of soluble vascular and intercellular cell adhesion molecules (sVCAM-1 and sICAM-1, respectively) and leukotriene B4, a potent pro-inflammatory factor [32]. In another study, the addition of 56 g/day of walnuts to the diet for eight weeks enhanced endothelial function in overweight adults with visceral obesity when compared with an ad libitum diet not supplemented with walnuts. As with almonds, although the walnut dose represented a large amount of calories, weight gain was not observed in the walnut treatment, which, curiously, was associated with a decline in waist circumference (WC) [28].

Overweight and visceral fat accumulation are known to be associated with low concentrations of adiponectin. The acute intake of a walnut-enriched meal improved postprandial adiponectin response in healthy young adults compared to butter- and olive oil-enriched meals. This result was accompanied with a smaller postprandial gene expression of tumor necrosis factor alpha (TNF-alpha) and interleukin (IL)-6 [33]. Likewise, short-term walnut consumption (48 g/day for four days) increased adiponectin concentration (15%) and improved the lipid profile, particularly Apo A concentrations [22].

The long-term inclusion of walnuts as part of a habitual diet favorably altered the plasma lipid profile. The supplementation of walnut in the amount of 12% of the total daily energy intake, without changing the habitual diet, during 12 months decreased TC and triglyceride concentrations. The beneficial effects of walnuts were more evident in subjects with higher baseline TC values. Thus, without adjusting for body weight, TC and triglyceride concentrations were significantly reduced in free-living individuals supplemented with walnuts when compared to a habitual diet. After adjusting for body weight, LDL-c was also reduced in a walnut-supplemented diet [24]. In a crossover study, replacement of 40% of the fat in the Mediterranean diet with walnuts, almonds, or virgin olive oil during four weeks each was associated with reductions in TC, LDL-c, and LDL-c/HDL-c. However, no changes were observed in HDL-c concentrations [34].

Walnuts contain antioxidants. A clinical trial tested acute and chronic consumption of 21 or 42 g/day of raw walnuts during six weeks and showed improvements in concentrations of red blood cells linoleic acid and pyridoxal phosphate only with chronic consumption, whereas total plasma thiols were enhanced acutely [23]. To compare the antioxidant effects of a meal containing walnut (81 g) or almond (91 g), with nuts providing 75% of the energy intake, Torabian et al. (2009) [35] conducted a study with healthy subjects. Plasma polyphenol concentration at 90 min was higher in walnut meal compared to almond meal. However, the plasma total antioxidant capacity at 150 min post-consumption of the nut meals was higher with the walnut meal compared to the almond meal. Both meals reduced the susceptibility of plasma to lipid peroxidation after 90 min of consumption [35].

Finally, Spaccarotella et al. showed that the consumption of 75 g/day of walnuts, rich in tocopherols, improved biomarkers of prostate and vascular status in men at risk for prostate cancer. It was also able to decrease the alpha and gamma-tocopherol ratio, increase the serum gamma-tocopherol, and trend towards an increase in the ratio of free prostate-specific antigen (PSA)/total PSA following eight weeks of supplementation, suggesting that walnuts improve biomarkers of prostate and vascular status [27]. Free PSA in combination with total PSA improves the specificity of diagnosis and decreases false positives.

Collectively, studies have observed a reduction of cardiovascular disease risk factors with walnut consumption of around 21–91 g/day for short- and long-term periods. Therefore, the consumption of walnuts may be related to the reduction of cardiovascular risk [27] both by improvement in lipid profile [22,25,34] and reduction of inflammatory and atherogenic processes [22,32,33]. The reduction of lipid peroxidation [35] and the improvement of endothelial function [24,28] are associated with high concentration of antioxidants present in walnuts [23,35], such as α-linolenic acid and alpha-tocopherol [3,26].

### 3.3. Pistachio

Table 3 summarizes the randomized studies regarding pistachio consumption [36,37,38,39,40,41,42,43,44]. Pistachio consumption has been associated with improvement of glucose metabolism [36,37], lipid profile [39,40], and vascular function [41,42]. In a clinical trial with mild dyslipidemic adults, regular consumption of shelled pistachios (40 g or 1.5 oz/day) for three months reduced fasting glucose and LDL-c concentrations and increased HDL-c with improvements in vascular function [41]. The pistachio (42 and 84 g/day) nut intervention during three weeks also lowered LDL-c concentrations (6%) in healthy volunteers [38].

Consumption of a larger amount of pistachios in the short term also yielded positive results in cardiovascular risk. Consumption of 57 g/day of pistachios in prediabetic subjects during eight weeks resulted in benefits in glucose metabolism and reductions in insulin resistance, inflammation, and several gene expressions, such as resistin, IL-6, fibrinogen, oxidized LDL-c, and platelet factor 4 [36].

Diabetic patients also benefit from the consumption of pistachios. The inclusion of two snacks with 25 g/day of pistachio nuts each was sufficient to decrease glycated hemoglobin (HbA1c), fasting blood glucose, systolic blood pressure, body mass index (BMI), and high-sensitivity C-reactive protein (hs-CRP) [37]. In another crossover study, well-controlled type 2 diabetes adults who received a diet containing 20% of their daily energy from pistachios for four weeks reduced TC, triglycerides, and fructosamines compared to a pistachio-free control diet [42]. The same intervention was conducted during 24 weeks with Asian Indians diagnosed with metabolic syndrome. The diet with pistachios demonstrated beneficial effects on the cardio-metabolic profile, such as reducing WC, LDL-c, free-fatty acids, hs-CRP, TNF-alpha, and thiobarbituric acid reactive substances (TBARS), and increasing adiponectin concentrations [39].

The chronic consumption of 57 g of pistachios daily shifted the lipoprotein size and particle profile to a less atherogenic pattern. Pistachios decreased small-LDL-c and HDL-c particle sizes, despite the absence of change in TC, LDL-c, or HDL-c concentrations. Thus, pistachios may play a beneficial role in cardiovascular disease independently of changes in the total plasma lipid profile [40].

Evaluation of the dose–response effect of pistachio intake (28, 56, and 84 g/day) alone or in combination with high-carbohydrate bread (50 g available carbohydrate) showed a reduction in post-prandial glycemia, highlighting a progressive reduction in bread glycemic response with the highest doses of pistachios [43]. Replacing 20% of the total energy from pistachios for four weeks modified systemic hemodynamics, increased heart rate variability, and reduced 24-h systolic blood pressure in adults with well-controlled type 2 diabetes [42]. However, the daily ingestion of either 42 g or 70 g/day of pistachios for 12 weeks caused no changes in the BMI or waist-to-hip ratio in Chinese subjects with metabolic syndrome [44]. Thus, clear evidence exists that pistachio consumption (range 25–84 g/day) may be beneficial to ameliorate the lipid profile [38,39,40,41] and attenuate the inflammatory markers [35,38] and blood pressure in individuals with overweight-associated risk factors [35,36]. Moreover, studies also show improvement in glucose metabolism [36,37,41,43] and improvement of vascular function and systemic hemodynamics [41,42].

### 3.4. Peanuts

As shown in Table 4, some studies have assessed associations between peanut consumption and postprandial blood glucose [45], nutrient intake [46], elevated fat oxidation rate [47], and increased satiety [48]. Despite portion size differences (42.5 to 63 g/day) and high energy content, there are no positive associations between peanut consumption and body fat. The consumption of large portions, reaching 70 g/day, did not result in changes in body composition [49].

The use of 46 g/day of peanuts and/or peanut butter in an American Diabetes Association (ADA) diet for 24 weeks increased the ratio of nutrients with cardioprotective properties (MUFA, PUFA, PUFA/saturated ratio, α-tocopherol, niacin, and magnesium) as compared to a peanut-free diet in adults with type 2 diabetes. The intervention suggested that the inclusion of peanuts and peanut butter was not obesogenic, because the body weight, BMI, and WC reduction did not differ from the peanut-free ADA meal plan [46].

Moreover, the inclusion of high-oleic peanuts in an energy-restricted diet improved fat oxidation and body composition in overweight subjects. Although the energy restriction was similar between groups that received or did not receive peanuts, those who consumed peanuts lost more weight than expected by energy restriction [47]. High-oleic peanuts, especially, increased diet-induced thermogenesis in overweight and obese men, supposedly by increasing the gene expression of uncoupling proteins (UCPs), in addition to reducing hunger through caloric compensation and increasing satiety [50]. Furthermore, high consumption of high-oleic peanuts (50–70 g/day) for 12 weeks, without energy restriction, did not increase body weight or WC [49].

Another randomized study evaluated the acute ingestion of 42.5 g/day of whole peanuts without skins and peanut butter added to a 75 g available carbohydrate-matched breakfast meal in obese women with high type 2 diabetes mellitus risk. Breakfast meals with whole peanut or peanut butter reduced the first-meal NEFA and the second-meal glycemic responses. In addition, they increased the peptide YY concentration, accompanied by a reduction in the desire to eat [48].

The ingestion of 63 g/day of ground roasted peanuts at breakfast also led to lower carbohydrate intake and reduced postprandial glycemic response, which might help improve glycemic control and reduce the diabetes risk. The study suggested that the cleavage of the cell walls after this processing method may release the fat content of the peanuts, resulting in the lower glycemic response observed [45]. Therefore, it is suggested that peanut consumption (42.5–75 g/day) in a period of three weeks, independent of body composition alteration, improves glycemic control, induces satiety, and attenuates NEFA concentration. Therefore, the inclusion of peanuts in the diet not only improves the quality of the diet [46], but also stimulates satiety [48], increases fat oxidation and thermogenesis [47], and reduces glycemic response [45]. Moreover, the consumption of peanuts in large portions (70 g) did not result in body weight gain [46].

### 3.5. Brazil Nuts, Hazelnuts, Cashew Nuts, and Macadamia

Brazil nuts are recognized by their antioxidant property, which is related to their selenium content. The addition of Brazil nuts to a diet could improve glutathione peroxidase activity [51,52] (Table 5). Consumption of one Brazil nut was effective in increasing the selenium status and the glutathione peroxidase activity in obese women regardless of the polymorphism-related genotype Pro198Leu [51]. In hypertensive and dyslipidemic individuals, ingestion of 13 g/day of Brazil nuts partially defatted reduced oxidative stress, oxidized-LDL-c and blood pressure, and increased antioxidant status [52].

Although findings with hazelnuts and cashew nuts suggest health benefits, clinical data have been limited. We found only one clinical trial with hazelnuts and one with cashew nuts (Table 5). Hazelnuts influence adherence and diet quality but not inflammatory markers or body composition in overweight and obese individuals. Nonetheless, the desire for and liking of nuts remained stable at 30 g/day, but the acceptability decreased over time with 60 g/day [53]. Regarding cashew nuts, the consumption of 28 to 64 g/day in mildly hypercholesterolemic adults decreased TC (−23.9% versus 4.5%) and LDL-c (−24.8% versus −3.1%), respectively, in comparison to a control diet [54]. The Brazil nuts antioxidant potential is one of the main benefits of their consumption for human health [51,52]. However, with regard to hazelnuts and cashew nuts, further studies are needed to elucidate their potential effects on human health, as well as the improvements on diet quality [53] and lipid profile [54].

## 4. Nutritional Composition of Nuts

The differences in the nutritional composition of nuts and peanuts are an important aspect that must be considered to elucidate the different effects of their intake on human health. Regarding lipid types, almonds, hazelnuts, and cashew nuts present a high proportion of MUFAs/saturated fatty acids (SFAs), with emphasis in hazelnuts, which present the highest proportion, corresponding to 10:1. Brazil nuts have the lowest MUFA/SFA ratio, and macadamias exhibit the highest MUFA/PUFA ratio. Walnuts have the highest level of PUFAs, mainly α-linolenic acid, corresponding to a total of 47.17 g/100 g of PUFAs, while almonds have the highest fiber of all the tree nuts, corresponding to 12.5 g/100 g. Peanuts, on the other hand, have a higher content of protein and fiber when compared to tree nuts. In addition to the different concentrations and combinations of fatty acids, it is important to emphasize that these foods also differ in micronutrients and bioactive substances, especially α-tocopherol in almonds and hazelnuts, selenium in Brazil nuts, phenolic compounds in walnuts, carotenoids in pistachios, and phytosterols in peanuts [3]. Thus, it is believed that nutritional characteristics can influence health effects as well as the choice of different portion sizes for prevention or coadjuvant treatments.

## 5. Practical Implications and Limitations

One serving of 10 g/day of almonds increased HDL-c concentrations in subjects with coronary disease, representing a protective factor for these patients [10]. On the other hand, a daily serving of 100 g of almonds resulted in a significant reduction in serum non-HDL-c concentrations in subjects on statin therapy [11]. The consumption of 60 g of almonds also provided a reduction of CT, LDL-c, and LDL-c/HDL-c and Apo B/Apo A-1 ratios [13].

One of the limitations of recommending larger portions of this food group is the fact that nuts are highly energetic. However, it is observed that almond intake does not stimulate body weight gain [11,19] and may aid in other aspects of human health, such as reduction of glycemic peaks and satiety control [12]. The absence of body weight gain could be explained by the overestimating of the metabolizable energy from almonds [18] walnuts [30], and pistachio [38]. The inclusion of walnuts in a regular diet is a good strategy to improve diet quality, which may result in health benefits in smaller (21 g) [23] and larger (75 g) portions [26], with potential action in reducing cardiovascular risk [27,55] and improving endothelial function [24,28].

Studies that evaluated the relationship between pistachios and human health used large portions compared to studies with almonds and walnuts. Daily portions of 57 g, 42 g, and 84 g of pistachios improved the glucose metabolism [36] and lipid profile [38]. The consumption of 40 g of pistachios appears to be effective in reducing fasting glucose concentrations and LDL-c, increasing HDL-c concentrations and improving vascular function [41]. In addition, the consumption of pistachios in larger portion sizes (70 g) was not related to weight gain [44].

Large portions of peanuts are recommended for their health benefits. Intervention portions range from 42.5 to 75 g/day [46,49]. However, despite the size of the portions offered in the studies and the high energy content of this food, there are no positive associations between peanut consumption and body fat, even in amounts of 70 g/day [49]. As a benefit to human health, peanuts can reduce postprandial blood glucose [45], improve micronutrient intake [46], and increase fat oxidation rate and satiety [47,48].

Brazil nuts have a large differential in relation to the portion size and effects on human health. One nut (approximately 5.0 g) is sufficient to increase serum selenium concentrations, as well as the activity of the enzyme glutathione peroxidase in obese women [51]. In hypertensive and dyslipidemic subjects, the intake of a larger portion contributed to reduced oxidative stress, LDL-c oxidation, and blood pressure [52].

Considering the scarcity of studies evaluating the relationship between the consumption of cashew nuts, macadamia, hazelnuts, and health outcomes, there are still questions regarding these nuts and their influence on human health. Ingestion of 30 g/day of hazelnuts did not result in reduction of some inflammatory markers; however, increasing the portion size of these foods becomes a challenge because there is low acceptance of portions containing 60 g of these foods [53].

Tree nuts and peanuts have been extensively investigated in individuals with cardiovascular diseases, with promising results for both prevention and treatment of associated comorbidities. However, studies that evaluate the consumption of these foods in patients with cancer and other pathologies with aspects of high oxidative and metabolic stress are still scarce. We found only one study that verified the effects of nuts in cancer, and the benefits were attributed to their antioxidant properties, especially of almonds [27].

Another aspect that should be taken into consideration is the allergenic potential of nuts [56]. Some studies have identified, for example, increased immunological sensitization by immunoglobulin E (IgE) through hazelnut ingestion, demonstrating a greater sensitivity to these foods and an allergic potential, especially among children [57]. Walnuts and peanuts may also have allergenic effects. However, thermal processing, such as roasting, can improve digestibility and reduce the behavior of allergenic proteins [58].

## 6. Conclusions

Tree nuts and peanuts have nutritional characteristics that can benefit human health, in particular, regarding the prevention and treatment of diseases. Due to the high energy density of nuts and seeds, it was believed that their consumption could increase weight gain; however, it is observed that the consumption of this group of foods does not stimulate weight gain. In contrast, the ingestion of nuts can help in the control of satiety and in the increase of thermogenesis. The introduction of these foods also promotes an increase in the quality of the diet, because they are rich in MUFAs, PUFAs, proteins, fibers, vitamins, minerals and bioactive compounds with antioxidant potential.

Moreover, nut intake demonstrates benefits on health outcomes, preventing and/or treating some chronic disease related risk factors, such as changes in glycemic and lipid metabolism, oxidative stress, and inflammation. However, further studies should be carried out to evaluate the effect of nuts on other pathologies, such as cancer and many kinds of inflammatory diseases.

## Figures and Tables

**Table 1:** Effects of almond consumption on health outcomes.

First Author (Year)	Number and Characteristics of Participants (M/F) and (Age)	Study Design (Length of the Intervention)	Control Group	Intervention Group(s)	Health Outcomes of Nut Consumption
Jamshed et al. (2015) [10]	150 CAD patients (113/37) (32–86 years)	Clinical trial (12 weeks)	Habitual diet without almonds	10 g/day of Pakistani almonds (PA) or American almonds (AA)	↑ HDL-c at 6 and 12 weeks in both AA and PA groups; ↓ TC, TG, LDL-c, VLDL-c, TC/HDL-c and LDL-c/HDL-c ratios, and atherogenic index at 6 and 12 weeks; No changes in BW and blood pressure.
Ruisinger et al. (2015) [11]	48 patients receiving statin (24/24) (18–78 years)	Randomized clinical trial (4 weeks)	Therapeutic Lifestyle Changes Diet counseling according to NCEP-ATPIII	100 g/day of almonds and Therapeutic Lifestyle Changes Diet counseling according to NCEP-ATPIII	↓ Non-HDL-c (4.9%); ↓ VLDL-c (3.3%); Shift from LDL-c pattern A to B particles; No changes in BW, TC, Lp (a), and HDL-c.
Tan et al. (2013) [12]	137 participants with increased risk for type 2 diabetes (48/89) (18–60 years)	Randomized, parallel-arm study (4 weeks)	Habitual diet without almonds	43 g/day of almonds with breakfast (BF) or lunch (LN), alone as a morning (MS) or afternoon (AS) snack	↓ Hunger, fullness, and desire to eat levels before the subsequent meal in all intervention groups. Hunger levels were suppressed more and remained below baseline when consumed as snacks (acute); ↓ AUC of glucose postprandial; ↑ MUFA e α-T ingestion; No changes in BW and fasting blood markers.
Li et al. (2011) [13]	20 Chinese with type 2 diabetes mellitus and with mild-hyperlipidemia (9/11) (58 ± 2 years)	Randomized crossover clinical trial (4 weeks/period) and 2 weeks of washout	NCEP-ATPIII: step II diet	60 g/day of almonds added to the control diet to replace 20% of total daily calorie intake	↑ Plasma α-T (26.8%); ↓ TC (6%), LDL-c (11.6%), LDL-c/HDL-c ratio (9.7%), Apo B (15.6%), Apo B/Apo A-1 ratio (16.7%), NEFA (5.5%), insulin (4.1%), fasting glucose (0.8%), and HOMA-IR (9.2%); ↓ Body fat (0.8%); ↑ PUFA, MUFA, fiber, Mg, and vitamin E ingestion.
Jenkins et al. (2008) [14]	27 hyperlipidemic men and women in postmenopausal stage (15/12) (48–86 years)	Randomized, crossover study (4 weeks/period) and >2 weeks of washout	Full dose of low-saturated fat (<5% energy) whole-wheat muffins	Full dose of almonds (73 ± 3 g/day) or half dose of almonds and half dose of muffins (mean 423 kcal/day)	↓ BW on half-dose group almonds; ↓ Malondyaldehide and creatinine-adjusted urinary isoprostane output in the full-dose almonds group; Higher urinary creatinine outputs and TC in both half and full dose; ↓ LDL-c and ↑ HDL-c in full-dose; No changes in α-T in both interventions.
Berryman et al. (2015) [15]	48 individuals with elevated LDL-c (22/26) (30–65 years)	Randomized, crossover study (6 weeks/period) and 2 weeks of washout	Diet with an isocaloric muffin substitution (no almonds) (58% CHO, 15% PRO, 26% total fat)	Cholesterol-lowering diet (51% CHO, 16% PRO, 32% total fat) with almonds (1.5 oz/day)	↓ TC (5.1 mg/dL), non-HDL-c (6.9 mg/dL), LDL-c (5.3 mg/dL), VLDL-c (2.31 mg/dL), Apo B (4.2 mg/dL), Apo B/Apo A1 (0.04), LDL-c/HDL-c (0.20), CRP (0.34 mg/dL); ↓ WC (0.8 cm), abdominal mass (0.19 kg), and fat mass; (0.07 kg) and leg fat mass (0.12 kg); No changes in BW.
Dhillon et al. (2016) [16]	86 overweight and obese adults (21/65) (18–60 years)	Randomized controlled clinical trial (12 weeks)	Nut-free diet with energy restriction (500-kcal deficit/day)	Almond-enriched diet (15% energy from almonds) with energy restriction (500-kcal deficit/day)	↓ Total (1.79%) and truncal (1.21%) fat, diastolic BP (2.71 mmHg), and a tendency to VAT (8.19 cm²) loss; No change in appetite rating and fasting serum blood profile.
Hollis et al. (2007) [17]	20 overweight and obese women (24 ± 9 years)	Randomized controlled crossover trial (10 weeks/period) and 3 weeks of washout	Habitual diet without almonds	Inclusion of 1440 kJ/day of almonds in the habitual diet	↑ Total fat, PUFA, MUFA, vitamin E, copper ingestion; ↑ Plasma α-T (21.6%); No changes in BW, resting metabolic rate, thermic effect of food, or total energy expenditure.
Gebauer et al. (2016) [18]	18 healthy individuals (10/8) (56.7 ± 2.4 years)	Randomized, crossover, controlled-feeding trial (3 weeks/period) and 1 week of washout	Typical American diet without almonds	42 g/day of almonds in different forms: whole natural almonds, whole dry roasted almonds, chopped almonds (dry roasted), and almond butter (dry roasted)	ME of whole natural almonds, whole roasted almonds, and chopped almonds was lower than predicted with Atwater factors; ME of whole natural almonds was lower than whole roasted almonds; ME of whole roasted and chopped almonds was lower than almond butter.
Foster et al. (2012) [19]	123 overweight and obese individuals (11/112) (18–75 years)	Randomized clinical trial (18 months)	Low-caloric diet	28 g/day packages of almonds (24 almonds per package) and low-caloric diet	↓ TG, TC, VLDL-c, TC/HDL-c, and TG at 6 months; ↓ BW at 6 months.
Dhillon et al. (2017) [20]	86 overweight and obese adults (21/65) (18–60 years)	Randomized clinical trial (12 weeks) or acute effect of a specific lunch	Nut-free control diet achieving 500 kcal/day or a high-CHO lunch (>85% energy from CHO)	Almond-enriched diet (AED) achieving 500 kcal/day or an almond-enriched high-fat lunch (A-HFL) (>55% energy from fat, almonds contributing 70–75% energy)	↑ Memory scores (57.7%) in A-HFL-group; ↓ Post-lunch dip in memory at a midday meal in both intervention groups.
Burns et al. (2016) [21]	29 parents (18–40 years) and 29 children (3–6 years; pairs)	Randomized controlled crossover trial (3 weeks/period) and 4 weeks of washouts	Habitual diet without almonds	1.5 oz/day of almonds for parents and 0.5 oz/day of almonds for children	↑ Total Healthy Eating Index score in parents and children (12.5% in both); Genus-level changes in microbiota occurred with nut intake, especially in children; ↑ Mg and vitamin E ingestion in parents and child.

Age was presented in mean ± standard deviation or range. M: male; F: female; ↓: reduction; ↑: increase; CAD: coronary artery disease; HDL-c: high-density lipoprotein cholesterol; TC: total cholesterol; TG: triglycerides; LDL-c: low-density lipoprotein cholesterol; VLDL-c: very low-density lipoprotein cholesterol; BW: body weight; NCEP-ATPIII: Third Report of the National Cholesterol Education Program Expert Panel on Detection, Evaluation, and Treatment of High Blood Cholesterol in Adults; Lp(a): lipoprotein A; ME: metabolizable energy; MUFA: monounsaturated fatty acids; α-T: alpha-tocopherol; Apo B: apoliprotein B; Apo A: apolipoprotein A; NEFA: non-esterified fatty acids; HOMA-IR: homeostasis model assessment; PUFA: polyunsaturated fatty acids; CHO: carbohydrate; PRO: protein; CRP: C-reactive protein; WC: waist circumference; BW: body weight; VAT: visceral adipose tissue.

**Table 2:** Effects of walnut consumption on health outcomes.

First Author (Year)	Number and Characteristics of Participants (M/F) and (Age)	Study Design (Length of the Intervention)	Control Group	Intervention Group(s)	Health Outcomes of Nut Consumption
Aronis et al. (2012) [22]	15 obese individuals (9/6) (58 ± 2.5 years)	Double-blinded, randomized, placebo-controlled, crossover study (4 days/period) and 4 days of washout	Isocaloric diet	48 g/day of walnuts + isocaloric diet	↑ Apo A and adiponectinina; No changes in fetuin A, resistin, CRP, serum amyloid A, ICAM1 e 3, VCAM1, IL-6 e 8, TNF-α, E-selectin, P-selectin, and thrombomodulin.
McKay et al. (2010) [23]	21 healthy individuals (postmenopausal) (9/12) (>50 years)	Randomized, crossover trial (6 weeks/period) and 6 weeks of washout	Habitual (control) diet	21 or 42 g/day of raw walnuts	↑ Red blood cell linoleic acid and plasma pyridoxal phosphate with 42 g/day; ↓ TC and LDL-c with 21 g/day; ↑ Total thiols within 1 h after walnut consumption with both doses of 21 and 42 g/day.
Ma et al. (2010) [24]	24 type 2 diabetes individuals (10/14) (30–75 years)	Single-blind, controlled, crossover study (8 weeks/period) and 8 weeks of washout	Ad libitum diet without walnuts	56 g/day (366 kcal) of walnuts and ad libitum diet	↑ Systolic/diastolic blood pressure (4.0/1.6 mmHg), insulin (3.6 mIU/mL); ↑ FBG (10.0 mg/dL); ↓ TC (9.7 mg/dL) and LDL-c (7.7 mg/dL); Improvement of endothelial function (FMD: 2.2%); No changes in anthropometric measurements, TG, HDL-c, HbA1c, and insulin sensitivity.
Torabian et al. (2010) [25]	87 normal to moderate high plasma total cholesterol adults (38/49) (30–72 years)	Randomized crossover design (6 months) and no washout	Habitual (control) diet	12% of total daily energy intake equivalent of walnuts (28–64 g/day)	↑ Red blood cell fatty acids, linoleic (1.2% mol) and α-linolenic acids (0.072% mol); ↓ TC (0.13 mg/dL) and TG (0.09 mg/dL), especially in subjects with high plasma TC.
Kranz et al. (2014) [26]	19 men at risk for developing prostate cancer (45–75 years)	Randomized controlled crossover trial (8 weeks/period) and 2 weeks of washout	Habitual (control) diet without walnuts	75 g/day of walnuts (490 kcal)	Higher energy intake, total fat, total fiber, MUFA, and PUFA ingestion; No changes in BW.
Spaccarotella et al. (2008) [27]	21 healthy men at risk for prostate cancer (45–75 years)	Randomized clinical trial (8 weeks)	Habitual (control) diet	75 g/day and usual diet of walnut supplement that was isocaloric incorporated in habitual diets	↑ TG peaked at 4 h, and γ-T at 4–8 h; ↑ γ-T (0.83 μmol/L); ↓ α-T (2.65 μmol/L), α-T/γ-T (3.49 μmol/L), and α-T/γ-T ratio adjusted for weight; ↑ Free PSA/total PSA, although PSA did not change.
Katz et al. (2012) [28]	46 overweight adults (18/28) (30–75 years)	Randomized controlled crossover trial, 2 (8 week/period) and 4 weeks of washout	Ad libitum diet without walnuts	56 g/day of walnuts and ad libitum diet	Improvement of endothelial function (FMD: 1.4%); No changes in BMI, body weight, WC, TC, LDL-c, HDL-c, TG, blood pressure, FBG, fasting, insulin, and HOMA-IR.
Tapsell et al. (2009) [29]	50 overweight adults with non-insulin-treated diabetes (no data) (35–75 years)	Randomized, parallel-group clinical trial (1 year)	Habitual (control) diet without walnuts	±30 g/day of walnuts targeting weight maintenance (around 2000 kcal, 30% fat) + low-fat dietary	↑ Total fat, PUFA, and protein ingestion; ↓ Weight, body fat, SAT; ↓ Leptin (all months), TC, LDL-c (6 months), HbA1c (3/6/9 months) insulin (3/6/9 months), FBG (3 months), and HOMA-IR (all months); ↑ HDL-c (6 months).
Baer et al. (2015) [30]	18 healthy adults (10/8) (25–75 years)	Dose–response, randomized, controlled, crossover trial (3 weeks/period) and 1 week of washout	Isocaloric food intake without walnuts	42 g/day or 84 g/day	The metabolizable energy of the walnuts was 21% less than that predicted by the Atwater factors in both intervention groups; ↑ Fecal wet weight, dry weight, and fat in fecal composition with 42 g/day walnut consumption.
Brennan et al. (2010) [31]	20 metabolic syndrome individuals (10/10) (40–75 years)	Randomized, double-blind, crossover study (4 days/period) and 2 weeks of washout	Shakes without nuts during the breakfast + isocaloric diet	Shakes during breakfast containing 48 g/day of walnuts in an isocaloric diet	↑ Satiety and sense of fullness in pre-lunch questionnaires; ↑ Area under curve PYY; No changes in resting energy expenditure and insulin resistance.
Canales et al. (2011) [32]	22 individuals at increased cardiovascular risk (no data) (men >45 and women >50 years)	Randomized, crossover study (5 weeks/period) and 4–6 weeks of washout	Lean meat	Walnut-enriched meat	↑ Paraoxonase activity; ↓ sICAM, aVCAM and leukotriene B4, paraoxonase-1/HDL-c, and paraoxonase-1/Apo A1 ratios; Paraoxonase levels negatively correlated with sICAM.
Lozano et al. (2013) [33]	21 healthy white men (18–30 years)	Randomized, double-blind, crossover study (1 day/period) and 1 week of washout	Meal with 60% fat, 15% protein, and 25% carbohydrates	Olive oil-enriched meal, butter-enriched meal, or walnut-enriched meal	↑ Adiponectin at 3 and 6 h after the walnut-enriched meal compared with the butter-enriched meal and higher at 6 h if compared with the olive oil-enriched meal; ↓ Free fatty acid from baseline at 3 h after the walnut-enriched meal.
Damasceno et al. (2010) [34]	18 hypercholeste-rolemic individuals (9/9) (58 ± 3 years)	Randomized, crossover (4 weeks/period) and no washout	Mediterranean diet	40 to 65 g/day of walnuts or 50 to 75 g/day of almonds or virgin olive oil (VOO—40% of the total fat and 22% of the total energy)	↓ LDL-c in all intervention groups, specifically, 7.3%, 10.8%, and 13.4% after the VOO, walnut, and almond diets, respectively; Higher plasmatic PUFA in walnut group.
Torabian et al. (2009) [35]	14 healthy individuals (7/7) (19–65 years)	Randomized, crossover study (3 weeks/period) and 1 week of washout	Control diet	Meal containing walnuts (81 g) or almonds (91 g) with nuts providing 75% of energy intake and the remaining 25% of energy from a refined carbohydrate source (polycose)	↑ Plasma polyphenol for both nuts (peak at 90 min), but walnut sustained higher concentration than almonds; Higher plasma total antioxidant capacity reached peak at 150 min post consumption of the nut meals, and higher after almonds than walnuts; ↓ Susceptibility to lipid peroxidation at 90 min for both walnut and almond.

Age was presented in mean ± standard deviation or range. M: male; F: female; ↓: reduction; ↑: increase; TC: total cholesterol; LDL-c: low-density lipoprotein cholesterol; FBG: fasting blood glucose; FMD: flow-mediated dilatation; TG: triglycerides; HbA1C: glycated hemoglobin; MUFA: monounsaturated fatty acids; PUFA: polyunsaturated fatty acids; BW: body weight; γ-T: gamma tocopherol; α-T: alpha tocopherol; PSA: prostatic-specific antigen; BMI: body mass index; WC: waist circumference; HDL-c: high-density lipoprotein cholesterol; HOMA-IR: homeostasis model assessment; VAT: visceral adipose tissue; PYY: peptide YY; Apo A: apolipoprotein A; ICAM: vascular cell adhesion protein; VCAM: vascular cell adhesion protein; IL- interleukin; TNF-α: tumor necrosis factor alpha.

**Table 3:** Effects of pistachio consumption on health outcomes.

First Author (Year)	Number and Characteristics of Participants (M/F) and (Age)	Study Design (Length of the Intervention)	Control Group	Intervention Group(s)	Health Outcomes of Nut Consumption
Hernández-Alonso et al. (2014) [36]	54 prediabetic subjects (29/25) (25–65 years)	Randomized, crossover study (8 weeks/period) and 2 weeks of washout)	No pistachio, normocaloric diet with 50% CHO, 15% PRO, and 35% total fat	57 g/day of pistachio and the same control diet	↓ FBG (5.17 mg/dL), insulin (2.04 mU/mL), and HOMA-IR (0.69); ↓ mRNA SLC2A4, IL-6, and resistin; ↑ Fibrinogen (2.24 ng/mL), oxidized-LDL-c (2.64 ng/mL), platelet factor 4 (0.07 ng/mL), and GLP-1 (4.09 pg/mL); ↑ Lutein-zeaxanthin (222.53 nmol/L) and γ-T (684.53 nmol/L).
Parham et al. (2014) [37]	48 diabetic individuals (11/37) (53 ± 10; 50 ± 11)	Double-blind, crossover study (12 weeks/period) and 8 weeks of washout	Control meal without nuts	Snack with 25 g pistachio nuts twice/day = 50 g/day of pistachio	↓ FBG (16 mg/dL) and HbA1C (0.4%).
Baer et al. (2012) [38]	18 healthy individuals (9/9) (29–64 years)	Randomized controlled crossover trial (3 weeks/period) and 2 weeks of washout	No pistachio	Pistachio doses were 42 g (1.5 oz/day) and 84 g/day (3.0 oz/day)	↑ Fecal wet weight in 1.5 oz/day, dry weight, fat, and energy in both intervention groups; ↓ Fat, energy, total CHO digestibility in both intervention groups; ↑ Total dietary fiber digestibility with 3.0 oz/day; ↓ LDL-c (6%) after both intervention groups.
Gulati et al. (2014) [39]	60 individuals with MS (37/31) (42.5 ± 8.2 years)	Randomized, double-blind control trial (24 weeks)	Normocaloric diet according to guidelines for Asian Indians	20% of total energy of normocaloric diet of pistachio/day	↓ WC (1.5 cm), FBG (3.9 mg/dL), TC (10.0 mg/dL), LDL-c (8.9 mg/dL), hs-CRP (0.8 mg/dL), TNF-α (3.7 pg/mL), FFA (34.2 µM), and TBARS (0.8 µM); ↑ Adiponectin (10.6 ng/mL).
Hernández-Alonso et al. (2015) [40]	54 prediabetic individuals (29/25) (55 years)	Randomized crossover clinical trial (4 months/period) and 2 weeks of washout	Control diet (55% CHO, 30% total fat)	57 g/day of pistachios (50% CHO and 33% total fat)	↓ sLDL-P; ↑ sHDL-P (2.23%); ↓ non-HDL-P (36.02 nM); Overall size of HDL-P was significantly lower (0.13 nM).
Kasliwal et al. (2015) [41]	56 mild dyslipidemia adults (46/10) (39.3 ± 8.1 years)	Randomized parallel-group study (3 months)	Lifestyle modification (LSM) alone	LSM with 80 g (in-shell) of pistachios (equivalent to 40 g or 1.5 oz shelled pistachios)	↑ HDL-c (2.1 mg/dL) ↓ LDL-c (9.6 mg/dL), TC/HDL-c ratio (0.5 mg/dL), and FBG (2.2 mg/dL); ↓ Left baPWV (27.7 cm/s).
Sauder et al. (2014) [42]	30 well-controlled type 2 diabetic adults (15/15) (40–74 years)	Randomized, crossover study (4 weeks/period) and 2 weeks of washout	Control meal without pistachio	20% of total energy of normocaloric diet of pistachio/day	↓ Systolic 24-h blood pressure (3.5 mmHg); ↓ TC/HDL-c ratio (3.7%), total peripheral resistance, and systolic 24-h blood pressure (3.5 mmHg); ↑ Cardiac output and improvement of some measurements of heart rate variability.
Kendall et al. (2011) [43]	10 healthy individuals (3/7) (48.3 ± 6.4 years)	Randomized, parallel-group clinical trial	White bread	Sudy 1: Dose–response effect of 28, 56, and 84 g/day pistachios consumed alone or co-ingested with white bread (50 g available carbohydrate); Study 2: 56 g/day of pistachio and carbohydrate foods (50 g available carbohydrate)	Dose–dependent reduction in the relative glycemic response in diet with CHO for both 56 and 84 g/day interventions; Pistachios consumed alone had a minimal effect on post-prandial glycemia.
Wang et al. (2012) [44]	90 metabolic syndrome individuals (41/49) (25–65 years)	Randomized controlled clinical trial (12 weeks)	No pistachios (DCG)	42 g/day pistachios (RSG) or 70 g/day pistachios (HSG)	↓ Glucose (1.13 mmol/L) after OGTT in HSG; ↓ TG (0.38 mmol/L) in RSG; ↑ LDL-c (0.31 mmol/L) in HSG; ↓ AST (7.81 U/L) in RSG and (5.52 U/L) in HSG.

Age was presented in mean ± standard deviation or range. M: male; F: female; ↓: reduction; ↑: increase; CHO: carbohydrate; PRO: protein; FBG: fasting blood glucose; HOMA-IR: homeostasis model assessment; SLC2A4: solute carrier family 2 member 4; IL: interleukin; LDL-c: low-density lipoprotein cholesterol; GLP1: glucagon-like peptide-1; HDL-c: high-density lipoprotein cholesterol; TC: total cholesterol; baPWV: brachial-ankle pulse wave velocity; HbA1C: glycated hemoglobin; MS: metabolic syndrome; WC: waist circumference; hs-CRP: high-sensible C-reactive protein; TNF-α: tumoral necrosis factor alpha; FFA: free fatty acids; TBARS: thiobarbituric acid reactive substances; OGTT: oral glucose tolerance test; TG: triglycerides; AST: aspartate transaminase; sLDL-P: small low-density lipoprotein particle; sHDL-P: small high-density lipoprotein particle; non-HDL-P: non high-density lipoprotein particle.

**Table 4:** Effects of peanut consumption on health outcomes.

First Author (Year)	Number and Characteristics of Participants (M/F) and (Age)	Study Design (Length of the Intervention)	Control Group	Intervention Group(s)	Health Outcomes of Nut Consumption
Reis et al. (2011) [45]	13 healthy individuals (4/9) (28.5 ± 10 years)	Randomized, crossover study (3 weeks/period) and 1 week of washout	No peanuts and cheese sandwich	63 g/day of raw peanuts with skin (RPS) or roasted peanuts without skin (RPWS) or ground-roasted peanuts without skin (GRPWS)	Improvement in glycemic response in RPS was higher than GRPWS; No changes in energy, macronutrients, and fiber ingestion.
Wien et al. (2014) [46]	60 type 2 diabetes individuals (30/30) (34–84 years)	Randomized, parallel-group clinical trial (24 weeks)	Peanut-free and ADA meal plan	±20% of energy from peanuts (46 g/day) in planned ADA meal	Higher PUFA/SFA diet ratio, MUFA, PUFA, α-T, niacin, and Mg ingestion; ↓ Weight, BMI, and WC; No changes in lipid profile.
Alves et al. (2014) [47]	65 overweight and obese men (18–50 years)	Randomized clinical trial (4 weeks)	Hypocaloric diet	Conventional peanuts (CVP) or high-oleic peanuts (HOP) that received the hypocaloric diet including (not adding) 56 g/day of peanuts	↓ Total fat mass in CVP and HOP; ↓ Gynoid fat in HOP; ↑ Total and gynoid lean mass in HOP; ↑ Fasting fat oxidation in CVP and HOP; ↑ Fat oxidation in HOP during 200 min after meal intake compared to fasting; ↓ Fullness in HOP.
Reis et al. (2013) [48]	15 type 2 diabetes and obese women (18–50 years)	Randomized, crossover study (1 day/period; acute) and 8 days of washout	No peanuts (NP)	42.5 g/day of whole peanuts without skins (WP) and peanut butter (PB) were added to a 75 g available CHO-matched breakfast meal	↑ Area under curve for NEFA (0–240 min) and glucose (240–490 min) for the PB breakfast; ↓ Second-meal glycemic response; ↑ Insulin in WP meal at 45 min and for the PB meal at 120 and 370 min for the PB breakfast; ↓ Appetite sensations and desire to eat in WP and PB; ↑ PYY, GLP-1, and CCK in PB; ↑ Fat consumption at WP; No changes in first-meal glycemic response in the none group.
Barbour et al. (2015) [49]	61 healthy individuals (29/32) (65 ± 7 years)	Randomized crossover design (12 weeks/period) and 6 weeks of washout	Nut-free diet	15–20% of energy/day of high-oleic peanuts	↑ Energy (10%) and fat intake, predominantly MUFA; No changes in body composition, and less than predicted increase (0.5 kg) in BW for the additional energy intake.

Age was presented in mean ± standard deviation or range M: male; F: female; ↓: reduction; ↑: increase; ADA: American Diabetes Association; PUFA: polyunsaturated fatty acids; SFA: saturated fatty acids; MUFA: monounsaturated fatty acids; α-T: alpha tocopherol; BMI: body mass index; WC: waist circumference; NEFA: non-esterified fatty acids; CHO: carbohydrate; PYY: peptide YY; GLP1: glucagon-like peptide-1; CCK: cholecystokinin; BW: body weight.

**Table 5:** Effects of Brazil nuts, hazelnuts, and cashew nuts consumption on health outcomes.

First Author (Year)	Number and Characteristics of Participants (M/F) and (Age)	Study Design (Length of the Intervention)	Control Group	Intervention Group(s)	Health Outcomes of Nut Consumption
Cominetti et al. (2011) [51]	37 morbidly obese women (reproductive age) (>18 years)	Randomized trial (8 weeks)	No nuts	One Brazil nut/day	↑ Plasma and erythrocyte selenium content; ↑ Glutathione peroxidase activity.
Huguenin et al. (2015) [52]	91 hypertensive and dyslipidemic subjects (47/44) (62.1 ± 9.3 years)	Randomized, crossover study (12 weeks/period) and 4 weeks of washout	Flavored cassava flour (10 g/day)	13 g/day of partially defatted Brazil nut and diet	↑ Plasma selenium (119%); ↑ Glutathione peroxidase activity (24.8%); ↓ Oxidized LDL-c (3.2%); An inverse association between glutatione peroxidase and oxidized LDL-c levels.
Tey et al. (2013) [53]	107 overweight and obese individuals (46/61) (18–65 years)	Randomized, double-blind, crossover study (12 weeks/period) and 2 weeks of washout	No nuts	30 g/day or 60 g/day of hazelnuts	Diet quality improvement in a dose–response manner. Desire and liking for nuts remained stable in the 30 g/day group, whereas these ratings ↓ Significantly over time in the 60 g/day group.
Mah et al. (2017) [54]	51 men and women (21–73 years)	Randomized, crossover study (28 days/period) and 2 weeks of washout	Potato chips (54% of CHO, 18% of PRO, and 29% of fat)	Cashews (28–64 g/day; 50% CHO, 18% of PRO, and 32% of fat)	↓ TC (3.9%), LDL-c (2.3%), TC/HDL-c ratio; No changes between diets for HDL-c cholesterol and TG.

Age was presented in mean ± standard deviation or range. M: male; F: female; ↓: reduction; ↑: increase; LDL-c: low-density lipoprotein cholesterol; TC: total cholesterol; HDL-c: high-density lipoprotein cholesterol; TG: triglyceride; CHO: carbohydrates; PRO: proteins.

## Abbreviations

ADA	American Diabetes Association
Apo A	apolipoprotein A
Apo B	apoliprotein B
AST	aspartate transaminase
baPWV	brachial-ankle pulse wave velocity
BMI	body mass index
BW	body weight
CAD	coronary artery disease
CCK	cholecystokinin
CHO	carbohydrate
CRP	C-reactive protein
FBG	fasting blood glucose
FFA	free fatty acids
FMD	flow-mediated dilatation
GLP1	glucagon-like peptide-1
HbA1C	glycated hemoglobin
HDL-c	high-density lipoprotein cholesterol
HOMA-IR	homeostasis model assessment
hs-CRP	high-sensible C-reactive protein
ICAM	vascular cell adhesion protein
IgE	immunoglobulin E
IL	interleukin
LDL-c	low-density lipoprotein cholesterol
Lp(a)	lipoprotein A
MDA	malondialdehyde
ME	metabolizable energy
MS	metabolic syndrome
MUFA	monounsaturated fatty acids
NCEP-ATPIII	Third Report of the National Cholesterol Education Program Expert Panel on Detection, Evaluation, and Treatment of High Blood Cholesterol in Adults
NEFA	non-esterified fatty acids
OGTT	oral glucose tolerance test
PRO	protein
PSA	prostatic-specific antigen
PUFA	polyunsaturated fatty acids
PYY	peptide YY
SFA	saturated fatty acids
sHDL-P	small high-density lipoprotein particle.
sLDL-P	small low-density lipoprotein particle
TBARS	thiobarbituric acid reactive substances
TG	triglycerides
TNF-α	tumor necrosis factor alpha
VAT	visceral adipose tissue
VCAM	vascular cell adhesion protein
VLDL-c	very low-density lipoprotein cholesterol
WC	waist circumference
α-T	alpha tocopherol
γ-T	gamma tocopherol
